# The importance of social activity to risk of major depression in older adults

**DOI:** 10.1017/S0033291721004566

**Published:** 2023-04

**Authors:** Euijung Ryu, Gregory D. Jenkins, Yanshan Wang, Mark Olfson, Ardesheer Talati, Lauren Lepow, Brandon J. Coombes, Alexander W. Charney, Benjamin S. Glicksberg, J. John Mann, Myrna M. Weissman, Priya Wickramaratne, Jyotishman Pathak, Joanna M. Biernacka

**Affiliations:** 1 Department of Quantitative Health Sciences, Mayo Clinic, Rochester, MN, USA; 2 Department of AI and Informatics, Mayo Clinic, Rochester, USA; 3 Department of Psychiatry, Columbia University and New York State Psychiatric Institute, New York, USA; 4 Department of Psychiatry, Icahn School of Medicine at Mount Sinai, New York, USA; 5 Mount Sinai Clinical Intelligence Center, Icahn School of Medicine at Mount Sinai, New York, USA; 6 The Hasso Plattner Institute for Digital Health at Mount Sinai, Icahn School of Medicine at Mount Sinai, New York, USA; 7 Department of Psychiatry, Weill Cornell Medicine, New York, USA; 8 Department of Psychiatry & Psychology, Mayo Clinic, Rochester, USA

**Keywords:** Biobank, depression, electronic health records, major depressive disorder, social activity, social determinants of health

## Abstract

**Background:**

Several social determinants of health (SDoH) have been associated with the onset of major depressive disorder (MDD). However, prior studies largely focused on individual SDoH and thus less is known about the relative importance (RI) of SDoH variables, especially in older adults. Given that risk factors for MDD may differ across the lifespan, we aimed to identify the SDoH that was most strongly related to newly diagnosed MDD in a cohort of older adults.

**Methods:**

We used self-reported health-related survey data from 41 174 older adults (50–89 years, median age = 67 years) who participated in the Mayo Clinic Biobank, and linked ICD codes for MDD in the participants' electronic health records. Participants with a history of clinically documented or self-reported MDD prior to survey completion were excluded from analysis (*N* = 10 938, 27%). We used Cox proportional hazards models with a gradient boosting machine approach to quantify the RI of 30 pre-selected SDoH variables on the risk of future MDD diagnosis.

**Results:**

Following biobank enrollment, 2073 older participants were diagnosed with MDD during the follow-up period (median duration = 6.7 years). The most influential SDoH was perceived level of social activity (RI = 0.17). Lower level of social activity was associated with a higher risk of MDD [hazard ratio = 2.27 (95% CI 2.00–2.50) for highest *v.* lowest level].

**Conclusion:**

Across a range of SDoH variables, perceived level of social activity is most strongly related to MDD in older adults. Monitoring changes in the level of social activity may help identify older adults at an increased risk of MDD.

## Introduction

Major depressive disorder (MDD) is a highly prevalent chronic condition both in the USA and worldwide, and it is estimated that one in six people will develop the disorder during their lifetime (Howard et al., 2019). The prevalence of MDD appears to be increasing over the past 25 years and age at first onset has been decreasing (Hasin et al., 2018; Sloan & Sandt, 2006). MDD is an important public health concern as it is associated with poor health, mortality, disability-years, functional impairment, and cognitive and social functioning, especially in the older population. Although first onset of MDD is less common in older adults compared to younger adults, with the first depression episode typically occurring before age 40 (Park et al., 2014; Sloan & Sandt, 2006), MDD (either late-onset or recurrent MDD) in older adults is fairly common and can lead to serious adverse consequences especially due to aging-related physical and cognitive impairment (Fiske, Wetherell, & Gatz, 2009). Given the rapid growth of older populations (Anderson, Goodman, Holtzman, Posner, & Northridge, 2012), it has become particularly important to identify those at risk for MDD episodes in order to reduce the personal and societal economic burden associated with the condition. Once identified, health care professionals can provide early, targeted interventions for those at risk for MDD by monitoring their symptoms before they develop MDD and ensuring they receive adequate treatment, which will be critical for reducing the burden of the disease in older adults. This is especially important, as compared to younger and middle-aged adults, older individuals with MDD are less likely to seek psychiatric treatment (~30% for 65+ *v.* ~45% for 35–54 years) (Mackenzie, Reynolds, Cairney, Streiner, & Sareen, 2012).

Numerous risk factors have been associated with MDD and the magnitude of association may differ across the lifespan (Emerson et al., 2018; Kendler, Gardner, & Prescott, 2002, 2006). For instance, familial risk is related more strongly to early-onset MDD (Kendler, Gatz, Gardner, & Pedersen, 2005). Furthermore, these associations may vary based on study design as a result of differences in study population, study sampling, diagnostic classification for MDD, and data source (e.g. surveys *v.* medical records). Nevertheless, well-documented MDD risk factors include genetics [the estimated heritability is between 30% and 40% (Power et al., 2017)], neurobiological factors (e.g. dysregulation of neurotransmitter systems such as serotonin), physical illness (e.g. cardiovascular diseases), and social determinants of health (SDoH; e.g. childhood abuse, lifetime adverse events, and lack of physical activities) (Sekhon, Patel, & Sapra, 2021). SDoH is defined as aspects of social environment that affect a wide range of health, functioning, and quality-of-life risk and outcomes (Andermann, 2016; Koh, Piotrowski, Kumanyika, & Fielding, 2011). Some examples of individual-level SDoH are socioeconomic status (SES), education, income, housing status, and social support networks (Singh et al., 2017). In MDD, the impact of SDoH is well documented: higher SES, the most commonly studied SDoH, has a protective impact on MDD, mainly due to the fact that people with higher SES are less likely to have certain adverse life events (e.g. trauma), health behaviors that are linked to adverse health, and have better healthcare access (Albert, 2016; Assari, 2017; Averina et al., 2005; Gibbs & Rice, 2016; Kim & Chen, 2011; Liang et al., 2012; McClintock & Bogner, 2017; Shittu et al., 2014; Tanner, Martinez, & Harris, 2014).

Beyond commonly studied SDoH (SES, income, and education), recent research has examined the role of more diverse SDoH variables, such as social support, on depression and found a strong contribution: depressed individuals with poorer social support and/or loneliness have worse outcomes in terms of symptom recovery or remission, and functional outcomes (Wang, Mann, Lloyd-Evans, Ma, & Johnson, 2018). A study of MDD using the UK Biobank found optimal sleep duration and lower screen time were protective against depressed mood (Sarris et al., 2020). Choi et al. reported that a higher level of physical activity was associated with a reduced risk of depression across all levels of genetic vulnerability (Choi et al., 2020). While SDoH (e.g. social support and adverse life events) in general plays a significant role in MDD at all ages, the degree of importance of specific types of SDoH may differ by age. For instance, SES and education play a stronger role in younger age groups (Kendler et al., 2005). However, the role of SDoH variables that act as a buffer for aging-related risk factors (e.g. cardiovascular diseases and cognitive impairment) is stronger in older adults and includes close social network, bereavement and living situation (Fiske et al., 2009; Litwin, Stoeckel, & Schwartz, 2015).

Despite the extensive literature on the associations between SDoH and mental health conditions, prior studies have typically analyzed each SDoH variable separately. However, these variables may interact with each other to contribute to the disease, and thus it is important to analyze different SDoH variables in mutually controlled models. In a population-based study using electronic health records (EHR) data, for example, higher SES and minority status (i.e. being other than non-Hispanic White) were associated with a lower risk of mood disorder, but the effect of minority status differed depending on SES, potentially due to issues such as healthcare access and literacy among individuals with lower SES (Wi et al., 2016). While a few studies have considered interactions among SDoH, these studies mostly investigated basic demographic characteristics (e.g. age, gender, race/ethnicity) and only a small number of other SDoH (e.g. education).

In the current study, we utilized data from the Mayo Clinic Biobank (MCB), including a health questionnaire with over 30 SDoH variables and linked EHR data that were used to identify participants with newly diagnosed MDD at Mayo Clinic in older age (50–89 years old at the time of enrollment in the MCB). We aimed to investigate multiple SDoH simultaneously to identify the most influential SDoH contributing to the development of MDD in older adults. Because the MCB sample is relatively homogeneous with regard to SES (~50% with college degrees or higher), self-reported race/ethnicity (~90% White), and geographic distribution, the findings may not generalize to all populations (Olson et al., 2013, 2019). Nevertheless, this study is an important step toward understanding the SDoH factors that impact MDD in the older adult populations that the MCB sample represents, which can subsequently be evaluated in other populations.

## Methods

### Study design and participants

This cohort study utilized data from Mayo Clinic patients who enrolled in the MCB. The design and governance of the MCB is described elsewhere (Hathcock et al., 2020; Olson et al., 2013, 2019). Briefly, the MCB started to enroll adults (age 18 or older) in April 2009 and ended active enrollment in March 2016. With some exceptions (i.e. volunteers who self-selected to participate without a study invitation), participants were largely selected through medical visits to primary care departments at Mayo Clinic. At consent, participants provided biological samples, completed a questionnaire, and provided permission for researchers to search their full EHR from all clinical visits (including past and future data) for studies approved by the MCB. This study was reviewed and approved by Mayo Clinic Institutional Review Board (IRB) and Mayo Clinic Biobank Access Committee.

At the time of pulling EHR data for the current study (6 April 2020), baseline survey data were available for a total of 41 174 participants who were 50–89 years old at the time of their biobank enrollment. We selected participants over 50 years of age at enrollment to study the role of SDoH in older adults, but excluded participants over 90 years old because they are unlikely to represent the general population in this age group [individuals with very poor physical/cognitive health, which are common in the oldest age group, are unlikely to travel to the clinic to enroll (Takahashi et al., 2013), even if they were able to provide informed consent to participate in the biobank]. Of those eligible, 10 938 (27%) participants had a known history of depression prior to the baseline survey completion, either documented in the EHR and/or self-reported on the enrollment questionnaire. Participants having at least one MDD-related ICD9/10 code in their EHR were considered as having a prior history of MDD in the EHR (see the Primary outcome section below). Self-reported depression was identified by a baseline survey question asking if a participant had been diagnosed with depression. Because our intent is to examine SDoH as potential risk factors for future MDD diagnosis, we excluded patients with a history of MDD and used the data for the remaining 30 236 participants to quantify variable importance of SDoH for the risk of MDD in older adults.

Although the MCB's recruitment strategy aimed to enrich for participants having comprehensive EHR, the MCB consists of participants with a wide range of EHR coverage. However, over 70% of the participants had clinic visits in at least 3 out of 5 years prior to consent or live in Mayo Clinic catchment areas (Olson et al., 2019). The study cohort has a median length of prior EHR records of 12.4 years (25th–75th percentiles: 4.2–30.6 years). To assess the impact of EHR coverage, we conducted a sensitivity analysis using only data from a subset of participants (*N* = 11 716) that are included in the Mayo Clinic Primary Care Panel (PCP; i.e. receiving regular primary care at Mayo Clinic).

### Primary outcome

The primary outcome of interest was a new MDD diagnosis since the time of biobank survey completion (index date). As described above, participants with a known history of depression (determined from the EHR or self-report) were excluded from the analysis. However, not all patients with an early or prior history of MDD may have been excluded because of either participants' failure to report or recall the disease which may have occurred many years before, or incompleteness of EHR to capture episodes from the past. Therefore, we label the main outcome as ‘new episode of MDD’, as opposed to ‘incident MDD’. Participants were followed up from index date until the last follow-up date (death date, the date of first MDD diagnosis, or 6 April 2020, whichever comes first). MDD was defined based on having at least one MDD-related ICD9/10 code, using an initial list of ICD9/10 codes mapped to phecodes for MDD [296.2 and 296.22, available from https://phewascatalog.org/phecodes (He et al., 2019; Wei et al., 2017)], with minor modification [adding dysthymic disorder (ICD9:300.4), depressive type psychosis (ICD9: 298.0), and atypical depressive disorder (ICD9: 296.82)]. The complete list of ICD9/10 codes is presented in online Supplementary Table S1.

### Primary predictors

The main goal of this study was to quantify the relative importance (RI) of SDoH variables in order to identify the most influential variables for the risk of new episodes of MDD in older adults. We selected, *a priori*, several SDoH variables collected from self-reported health questionnaires administered at the time of biobank enrollment, including (a) perceived level of social activity (low, medium, high); (b) six questions from ENRICHD Social Support Instrument (ESSI; someone available to listen, give advice, show love/affection, help with daily chores, provide emotional support, to trust/confide) (Vaglio et al., 2004); (c) general health behaviors (e.g. smoking status); (d) physical activities (e.g. exercise); and (e) environmental variables (e.g. secondhand smoking). In addition, we also considered several demographic variables (e.g. age, gender, race/ethnicity, education attainment, and marital status). Age at time of enrollment in the biobank and gender information were extracted from EHR, while the rest of the SDoH data were collected from the survey. The survey questions are listed in online Supplementary Table S2. The ESSI questions measure different aspects of social support that are correlated. For a secondary analysis, we also calculated a perceived social support score using five of the aforementioned six ESSI questions (omitting ‘someone to help with daily chores’) using a previously established approach (Gan et al., 2019). Scores of the five questions were summed (ranging from 5 to 25), and dichotomized to determine the degree of social support (low *v.* high): Low social support was defined as a total score ⩽18 and a score ⩽3 (none, little, or some of the time) for at least two questions (Gan et al., 2019).

### Statistical analysis

Descriptive statistics [median (25th–75th percentile) for continuous variables and percentage for categorical variables], were used to characterize the study sample. For most variables, the percentage of missing information was <1% with some exceptions such as exercise questions (4% missing). The main goal of the analysis was to quantify the RI of SDoH variables when analyzing 30 variables simultaneously. Analysis limited to participants with complete data (which would exclude ~20% of the participants) may lead to biased results, and thus we first imputed missing SDoH data using Multiple Imputation by Chained Equation (MICE) within a random forest framework (Shah, Bartlett, Carpenter, Nicholas, & Hemingway, 2014) with five repetitions, using the R package *mice* v3.10-0. Following imputation, the univariate association between each SDoH variable and the risk of MDD was tested using a Cox regression model adjusting for age (using a quadratic spline) and gender in each of the five imputation replicates. For each SDoH variable, the model parameter estimates and test statistics [hazard ratio (HR), 95% confidence interval (CI) and associated *p* values] from the five repetitions were aggregated using ‘Rubin's Rules’, implemented in the R package *miceadds* v3.10–28.

To quantify the RI of each SDoH variable for predicting MDD, we used Cox proportional hazards models with gradient boosting machine (GBM) approach (Natekin & Knoll, 2013). The analysis was performed for each imputation replicate and the median of RIs from the five imputation repetitions was used to identify the most influential variable. The GBM models were constructed using the R package *gbm* v2.1–8 with 80% of the data used as the training dataset, with 25 000 trees, fivefold cross-validation, 100% bag fraction, and two-way interactions included. Using the final fitted model, the RI of each SDoH variable was calculated using a feature importance ranking measure on quantifying the ‘flatness’ of the effects of each variable on the risk of MDD, assessed by partial dependence plots (PDPs) (Greenwell, Boehmke, & McCarthy, 2018) using the R package *pdp* v0.7-0. Relative ‘flatness’ was defined by the standard deviation of predicted partial dependent function over the data range of each SDoH variable, with a higher score implying greater RI.

After quantifying the RI of each SDoH variable, potential interaction effects between the most influential variables and the rest of the SDoH were tested using Cox regression models, adjusting for age and gender. We also conducted a sensitivity analysis by repeating the entire analysis using only data from participants who were included in the PCP at the time of biobank enrollment. All analyses were repeated using an aggregated social support question (dichotomized), instead of using the five separate ESSI social support questions described above.

## Results

### Cohort characteristics

After excluding participants who had a prior history of depression identified via either EHR or self-report, a total of 30 236 patients aged 50–89 years at the time of enrollment were included in the study. The study cohort had a median age at the index date of 67 years, was about half female, mostly white (93%), non-Hispanic (98%), and US born (95%). Over 50% had a 4-year college degree or higher (Table 1). Low social connection was reported by 11% of the participants for the level of social activity and 16% for social support (Table 2). Roughly 6% of the participants reported having little to no time with someone they trust and confide in (online Supplementary Table S3). During the follow-up (median follow-up duration: 6.7 years), 2073 (6.8%) participants had at least one diagnosis code for MDD.

**Table 1.** Basic characteristics of the study participants and univariate association results (adjusted for age and gender) between each characteristic and risk of major depressive disorder

Characteristics	Summary (*N* = 30 236)	Hazard ratio (95% CI)	*p* value
Age (in years) at survey			
Median (25th−75th %tile)	67 (59–74)	–	–
Gender, *n* (%)			
Female	15 191 (50.2%)	–	–
Male	15 045 (49.8%)	–	–
Race, *n* (%)			
White	28 139 (93.1%)	0.85 (0.72–1.00)	0.063
Others	2097 (6.9%)	Ref	
Hispanic, *n* (%)			
Yes	340 (1.1%)	1.46 (1.02–2.09)	0.054
No	29 551 (97.7%)	Ref	
*Missing*	345 (1.1%)		
US born, *n* (%)			
Yes	28 841 (95.4%)	1.01 (0.81–1.25)	0.945
No	1283 (4.2%)	Ref	
*Missing*	112 (0.4%)		
Marital status, *n* (%)			
Married	24 334 (80.5%)	Ref	<0.001
Marriage-like relationship	726 (2.4%)	1.23 (0.94–1.62)	
Separated/divorced	1724 (5.7%)	1.37 (1.15–1.62)	
Widowed	1983 (6.6%)	1.24 (1.05–1.46)	
Never been married	876 (2.9%)	1.11 (0.87–1.42)	
*Missing*	593 (2.0%)		
Educational attainment, *n* (%)			
High school or less	5431 (18.0%)	Ref	<0.001
Some college	8910 (29.5%)	0.89 (0.79–1.00)	
College graduate	7215 (23.9%)	0.62 (0.54–0.71)	
Graduate/professional degree	8189 (27.1%)	0.66 (0.58–0.75)	
*Missing*	491 (1.6%)		
Employment, *n* (%)			
Full-time	8981 (29.7%)	Ref	<0.001
Part-time	3484 (11.5%)	1.19 (1.01–1.40)	
Not working for pay	17 322 (57.3%)	1.34 (1.18–1.53)	
*Missing*	449 (1.5%)

**Table 2.** Univariate association results (adjusted for age and gender) between social connection (activity and support) questions and risk of major depressive disorder in the study cohort

Characteristics	Summary (*n* = 30 236)	1-year MDD rate (%; 95% CI)	Hazard ratio (95% CI)
Perceived level of social activity, *n* (%)			
Low	3422 (11.3%)	3.7% (3.1%–4.4%)	Ref
Medium	6233 (20.6%)	1.3% (1.1%–1.7%)	0.58 (0.50–0.66)
High	20 514 (67.8%)	0.9% (0.8%–1.0%)	0.44 (0.40–0.50)
*Missing*	67 (0.2%)		
Perceived social support^a^, *n* (%)			
Low	4776 (15.9%)	1.8% (1.5%–2.3%)	Ref
High	25 296 (84.1%)	1.2% (1.1%–1.4%)	0.74 (0.67–0.83)
*Missing*	164 (0.5%)		

a Perceived low social support is a composite score for measuring perceived social support, using five ENRICHD Social Support Instrument (ESSI) questions (someone to listen, someone to give advice, someone for love, someone for emotional support, and someone to trust and confide).

### Univariate associations between SDoH and MDD risk

**Fig. 1.**
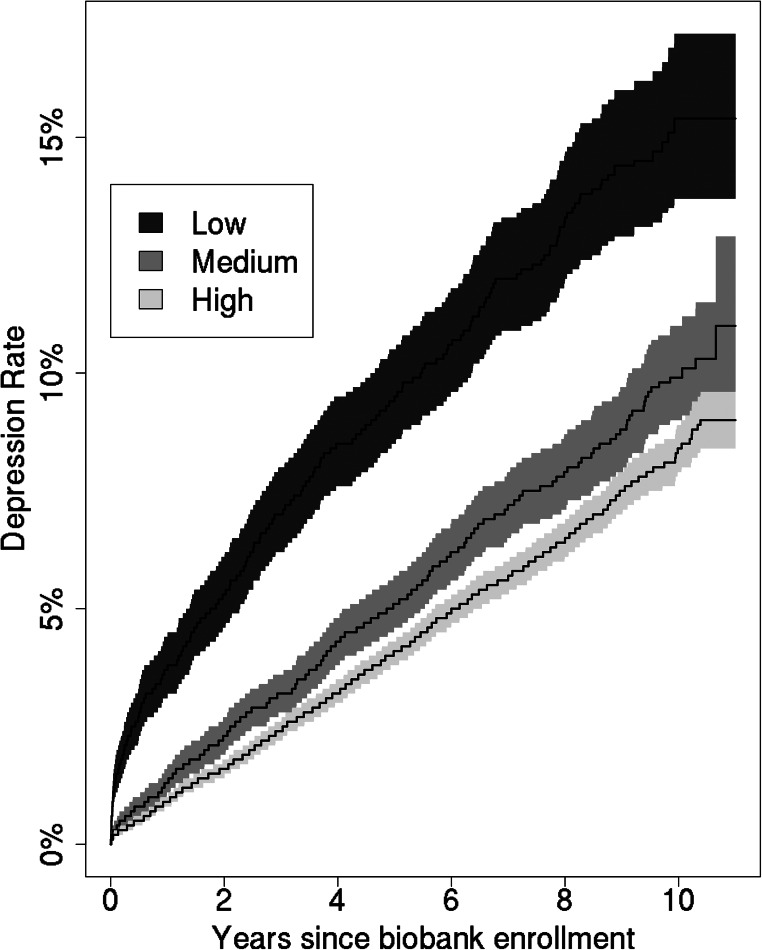
Kaplan–Meier plot for developing major depressive disorder, by perceived level of social activity.

Participants in their 60s had the lowest rate of new diagnoses of MDD and the risk increased with advancing age. MDD risk was higher among women than men [HR = 1.37 (95% CI 1.25–1.49)]. Most of the SDoH variables (collected at the index date, prior to MDD diagnosis) were associated with the risk of MDD (Tables 1 and 2, and online Supplementary Table S3). For instance, after adjusting for age and gender, a higher level of social activity was associated with a reduced risk of MDD [HR = 0.44 (95% CI 0.40–0.50) when comparing participants having high level *v.* low level; see Table 2 and Fig. 1]. Similarly, participants with high social support also had a lower risk of MDD compared to those with low support [HR = 0.74 (95% CI 0.67–0.83)], although the effect size was smaller than for social activity. Similar findings were observed in the sensitivity analysis restricted to the subset of the cohort consisting of participants who were included in the Mayo Clinic PCP (i.e. those who received usual medical care at Mayo Clinic) at the time of biobank enrollment (online Supplementary Table S3).

### Relative importance of SDoH on MDD risk

**Fig. 2.**
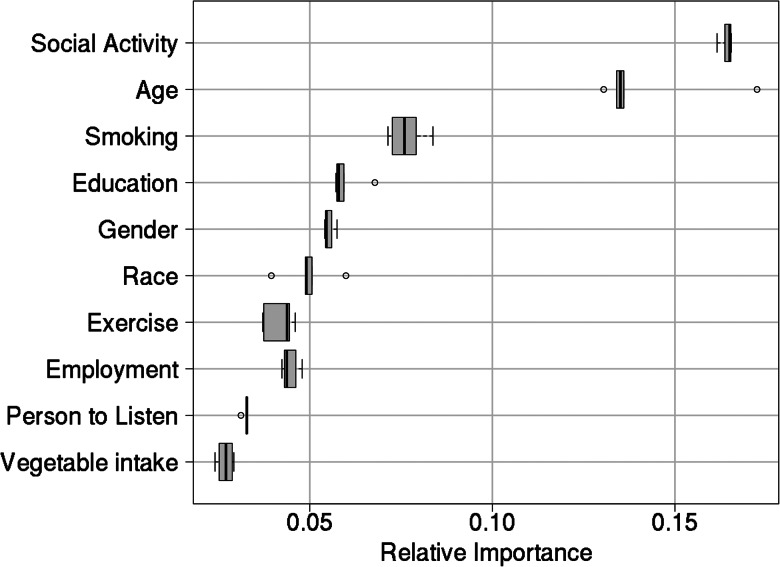
Relative influence of the top 10 social determinants of health variables for the risk of major depressive disorder.

**Fig. 3.**
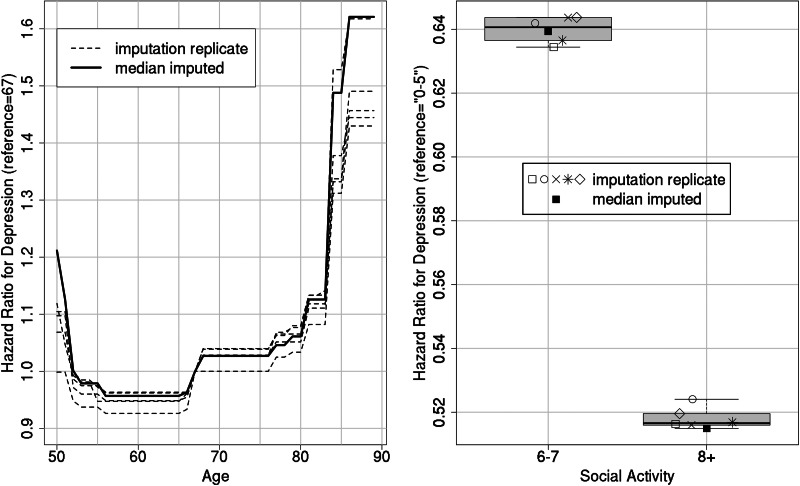
Relationship of age (left panel: comparing to the median age of the cohort) and perceived level of social activity (right panel: comparing with low perceived level of social activity [0–5]) with the risk of major depressive disorder.

Among the 30 SDoH variables considered (including age and gender), the most influential variable for the risk of a new episode of MDD was the level of social activity (median RI = 0.17; Fig. 2), followed by age (median RI = 0.14). Overall, MDD risk after age 60 years increased with age, which may indicate late-onset MDD. Higher level of social activity was associated with a lower risk of MDD (Fig. 3). ESSI social support questions had a much smaller influence (median RI scores <0.03; online Supplementary Fig. S1). A separate analysis showed that the influence of social support (as a composite score, rather than using individual ESSI variables) was also small compared with the effect of the level of social activity (data not shown). In a sensitivity analysis restricted to participants who received usual medical care at Mayo Clinic (PCP cohort), the relationship of the level of social activity to MDD risk was also strong (although age had a stronger influence in this sub-cohort) and effect sizes were similar (online Supplementary Figs. S1 and S2).

### Interaction between the level of social activity and other SDoH

To assess whether the most influential SDoH variable for the risk of a new episode of MDD (level of social activity) interacted with other SDoH to contribute MDD risk, we tested for interaction effects between the level of social activity and other SDoH, adjusting for age and gender. None of the other SDoH variables interacted with the level of social activity (all interaction *p* values ⩾0.05), implying that the influence of level of social activity on MDD risk was independent of age (interaction *p* = 0.42), gender (interaction *p* = 0.36), and social support (e.g. interaction *p* = 0.75 for the availability of someone to trust and confide in). The results were similar for the PCP cohort (online Supplementary Table S4).

## Discussion

This study quantifies the RI of various SDoH to the risk of a new episode of MDD in older adults. In addition to age (a known risk factor for MDD), perceived level of social activity was the SDoH most closely correlated with a new diagnosis of MDD. The effect of level of social activity was independent of other SDoH such as age, gender, and social supports. Further studies are needed for replication and to assess whether these findings reflect SDoH being a consequence of depression or a cause of depression. Given the lack of diversity in the study population, further studies are needed to determine if the findings also hold for more diverse populations, such as other ethnic groups. However, given that the level of social activity can be assessed via a single question, it could be easily implemented in routine clinical practice as a means of screening older adults for MDD risk followed by more detailed mental health assessments of individuals with low level. Assessing patients' perceptions about their level of social activity (or related psychological states such as loneliness) is important because it may be difficult for primary care clinicians to identify patients with few social connections (Due, Sandholdt, Siersma, & Waldorff, 2018). This aspect is particularly important in the COVID-19 pandemic era where social isolation and depressive symptoms have become more prevalent (Bu, Steptoe, & Fancourt, 2020; Vahratian, Blumberg, Terlizzi, & Schiller, 2021).

A large volume of literature demonstrates the importance of SDoH as risk factors for mental health conditions including depression. Fiske et al. suggested that different SDoH variables contribute to depression over the life span: for instance, high education plays a protective role for depression in young adults. However, social connection such as engagement and a close social network emerge as protective factors for depression in the elderly, which is likely because these factors act as a buffer for aging-related risk factors (e.g. physical/cognitive impairment and cardiovascular disease) that are specific to older adults (Fiske et al., 2009). As an example, Roy et al. reported that adverse effects of living alone (an important SDoH in the elderly) on psychological stress were not present among older adults with greater social participation (Roy et al., 2018). The importance of social connections is further supported by a study based on adults aged 57–85 years, in which social disconnectedness (e.g. small social network, infrequent social interaction) predicted higher subsequent perceived social isolation (e.g. loneliness, lack of support), which in turn predicted higher depression symptoms (Santini et al., 2020). Our finding that the level of social activity (an aspect of social connection) is the most influential SDoH in older adults is in line with these findings.

Several studies have reported associations between social activity and mental health conditions. Participation in social activities may protect against depression in elderly patients, due to stimulating bodily systems, and reinforcing life-long patterns of attachment (Lee & Kim, 2014). However, the association may differ by types of activity and culture. A study based on older adults from 10 European countries showed that social activity was associated with depressive symptoms, but the association direction and strength depended on the types of social activities, with participation in religious organizations having greater benefits than other forms of social activities (e.g. volunteer work), which may be partly because religious participation may provide a coping mechanism and prevent social isolation (Croezen, Avendano, Burdorf, & van Lenthe, 2015). However, a longitudinal study based on older Asian adults showed that attending religious services was related to an increase in depressive symptoms among those who were not depressed at baseline, whereas participating in social gatherings with friends and neighbors was associated with a decrease in depressive symptoms (Min, Ailshire, & Crimmins, 2016). Flatt et al. also emphasized the importance of considering different types of social activities in relation to cognitive health and general well-being in older adults (Flatt et al., 2015). Therefore, inconsistent results regarding the association between social activity and mental health may be related to differences in types of social activities and cultural context. As a measure of social activity, we used the perceived level of social activity that incorporates an individual's own evaluation, which is less likely to be affected by the types of activity, frequency, and study setting.

This study has several strengths and limitations. As a strength, our study used data from the MCB; a large portion of this cohort receive primary care at Mayo Clinic and thus have comprehensive EHR data (over 25 years since 1994) available for research (Olson et al., 2019). In addition, our study used both longitudinal EHR and biobank-collected survey data that included SDoH-related questions in addition to medical history. Because SDoH information is not routinely collected in clinical care and therefore not available in EHR, these data provide a unique opportunity to study the role of SDoH on various health outcomes including mental health conditions.

Our study also has several limitations. First, the cohort may not represent the local population because of selection bias that results from enrollment into the MCB. On average, the older participants (50+) are likely in poorer health than their age-matched counterparts from the general population because recruitment was largely based on medical appointments, but might be healthier in the oldest age group because individuals with very poor physical/cognitive health, which is common in this age group, are unlikely to travel to the clinic to enroll (Takahashi et al., 2013). Because such selection bias might pose the greatest threat to this oldest age group, we excluded biobank participants who were 90 years or older from the analysis. Second, the current study used ICD-9/10 diagnostic billing codes from structured EHR data as the source of case ascertainment, as opposed to other standard approaches such as standardized interviews (First, 2015). Therefore, misclassification of MDD (especially false negatives) based on ICD codes may be high. Additionally, it is well understood that mental health conditions including depression are generally under-coded in EHR. The concordance between clinical major depression diagnoses and independent assessments is fair to modest (Townsend, Walkup, Crystal, & Olfson, 2012). Also, patients who receive medical care at Mayo Clinic can also seek medical care, including psychiatric care, from other medical centers where the current study was unable to use the data. Nevertheless, EHR-based case ascertainment facilitates efficient large-scale studies compared to traditional approaches and has been used successfully in studies of numerous complex traits including psychiatric disorders (Chen et al., 2018; Li, Chen, Ritchie, & Moore, 2020; Smoller, 2018). Third, our study sample did not capture the period when depression prevalence is highest, before age 40 years. As a consequence, some participants included in our study, despite having no clinically diagnosed MDD in their EHR and no self-reported depression at the index date, may still have had a history of depression that was missed due to incomplete EHR and recall bias in self-report. Therefore, our findings may partly reflect the consequences of depression, rather than risk factors for depression, as it is possible that some participants reporting a low level of social activity may already have been experiencing depressive symptoms. Fourthly, there are other factors such as medical comorbidities, cognitive impairment, and activities of daily living that are associated with depression and therefore may have affected the study findings. However, this information was not evaluated in the biobank questionnaire (the source of the SDoH data in this study) and thus was not included in our study. Lastly, our study results may not be generalizable to a more diverse population in terms of race/ethnicity and education, and our study may not have had sufficient statistical power for assessing the importance of some SDoH variables that had low frequencies in our sample including ethnic/racial minority ancestry. As another example, educational attainment is a well-known strong SDoH associated with numerous health outcomes. However, its importance was not strong compared to social activity in our study of older adults. Although it is possible that education may not be as influential on depression in later life, it is also possible that our study lacked statistical power to detect the effect of education due to the overall high level of educational attainment in the study cohort (18% of the MCB participants with high school degree or lower, compared to 34% of the 2010 US population).

In conclusion, our study identified perceived level of social activity as the most influential SDoH variable in older adults. As this variable is easily measurable (as a single question) and its influence is independent of demographic characteristics and other SDoH variables, it could be easily implemented in clinical care to identify patients with an elevated risk of depression who could then be targeted for early intervention.

## Supplementary material

For supplementary material accompanying this paper visit https://doi.org/10.1017/S0033291721004566. click here to view supplementary material

## References

[ref1] Albert, S. M. (2016). Social determinants of geriatric depression. The American Journal of Geriatric Psychiatry, 24(12), 1209–1210. doi: 10.1016/j.jagp.2016.09.002 27717680

[ref2] Andermann, A. (2016). Taking action on the social determinants of health in clinical practice: A framework for health professionals. Canadian Medical Association Journal, 188(17–18), E474–E483. doi: 10.1503/cmaj.160177 27503870 PMC5135524

[ref3] Anderson, L. A., Goodman, R. A., Holtzman, D., Posner, S. F., & Northridge, M. E. (2012). Aging in the United States: Opportunities and challenges for public health. American Journal of Public Health, 102(3), 393–395. doi: doi:10.2105/Ajph.2011.300617 22390500 PMC3487684

[ref4] Assari, S. (2017). Social determinants of depression: The intersections of race, gender, and socioeconomic status. Brain Sciences, 7(12), 156. doi: 10.3390/brainsci7120156 PMC5742759 29186800

[ref5] Averina, M., Nilssen, O., Brenn, T., Brox, J., Arkhipovsky, V. L., & Kalinin, A. G. (2005). Social and lifestyle determinants of depression, anxiety, sleeping disorders and self-evaluated quality of life in Russia – a population-based study in Arkhangelsk. Social Psychiatry and Psychiatric Epidemiology, 40(7), 511–518. doi: 10.1007/s00127-005-0918-x 16088370

[ref6] Bu, F., Steptoe, A., & Fancourt, D. (2020). Who is lonely in lockdown? Cross-cohort analyses of predictors of loneliness before and during the COVID-19 pandemic. Public Health, 186, 31–34. doi: 10.1016/j.puhe.2020.06.036 32768621 PMC7405905

[ref7] Chen, C. Y., Lee, P. H., Castro, V. M., Minnier, J., Charney, A. W., Stahl, E. A., … Smoller, J. W. (2018). Genetic validation of bipolar disorder identified by automated phenotyping using electronic health records. Translational Psychiatry, 8, 86. doi: 10.1038/s41398-018-0133-7 PMC5904248 29666432

[ref8] Choi, K. W., Zheutlin, A. B., Karlson, R. A., Wang, M. J., Dunn, E. C., Stein, M. B., … Smoller, J. W. (2020). Physical activity offsets genetic risk for incident depression assessed via electronic health records in a biobank cohort study. Depression and Anxiety, 37(2), 106–114. doi: 10.1002/da.22967 31689000 PMC7905987

[ref9] Croezen, S., Avendano, M., Burdorf, A., & van Lenthe, F. J. (2015). Social participation and depression in old age: A fixed-effects analysis in 10 European countries. American Journal of Epidemiology, 182(2), 168–176. doi: 10.1093/aje/kwv015 26025236 PMC4493978

[ref10] Due, T. D., Sandholdt, H., Siersma, V. D., & Waldorff, F. B. (2018). How well do general practitioners know their elderly patients’ social relations and feelings of loneliness? BMC Family Practice, 19, 34. doi: 10.1186/s12875-018-0721-x PMC5828068 29482509

[ref11] Emerson, N. D., Small, G. W., Merrill, D. A., Chen, S. T., Torres-Gil, F., & Siddarth, P. (2018). Behavioral risk factors for self-reported depression across the lifespan. Mental Health & Prevention, 12, 36–41. doi: 10.1016/j.mhp.2018.09.002

[ref12] First, M. B. (2015). Structured clinical interview for the DSM (SCID). In R. L. Cautin & S. O. Lilienfeld (Eds.), The Encyclopedia of Clinical Psychology. doi: 10.1002/9781118625392.wbecp351

[ref13] Fiske, A., Wetherell, J. L., & Gatz, M. (2009). Depression in older adults. Annual Review of Clinical Psychology, 5, 363–389. doi: 10.1146/annurev.clinpsy.032408.153621 PMC2852580 19327033

[ref14] Flatt, J. D., Hughes, T. F., Documet, P. I., Lingler, J. H., Trauth, J. M., & Albert, S. M. (2015). A qualitative study on the types and purposes of social activities in late life. Activities Adaptation & Aging, 39(2), 109–132. doi: 10.1080/01924788.2015.1024485 PMC4727247 26823639

[ref15] Gan, Y. X., Xiong, R., Song, J. J., Xiong, X. L., Yu, F., Gao, W. M., … Chen, D. (2019). The effect of perceived social support during early pregnancy on depressive symptoms at 6 weeks postpartum: A prospective study. BMC Psychiatry, 19, 232. doi: 10.1186/s12888-019-2188-2 PMC6664519 31357958

[ref16] Gibbs, J. J., & Rice, E. (2016). The social context of depression symptomology in sexual minority male youth: Determinants of depression in a sample of Grindr users. Journal of Homosexuality, 63(2), 278–299. doi: 10.1080/00918369.2015.1083773 26295497

[ref17] Greenwell, B. M., Boehmke, B. C., & McCarthy, A. J. (2018). A simple and effective model-based variable importance measure. arXiv:1805.04755. Retrieved from https://ui.adsabs.harvard.edu/abs/2018arXiv180504755G.

[ref18] Hasin, D. S., Sarvet, A. L., Meyers, J. L., Saha, T. D., Ruan, W. J., Stohl, M., & Grant, B. F. (2018). Epidemiology of adult DSM-5 major depressive disorder and its specifiers in the United States. JAMA Psychiatry, 75(4), 336–346. doi: doi:10.1001/jamapsychiatry.2017.4602 29450462 PMC5875313

[ref19] Hathcock, M. A., Kirt, C., Ryu, E., Bublitz, J., Gupta, R., Wang, L., … Olson, J. E. (2020). Characteristics associated with recruitment and re-contact in Mayo Clinic Biobank. Frontiers in Public Health, 8, 9. doi: 10.3389/fpubh.2020.00009 32117849 PMC7010638

[ref20] He, M. D., Ortiz, A. J. S., Marshall, J., Mendelsohn, A. B., Curtis, J. R., Barr, C. E., … Kim, S. C. (2019). ICD-9 to ICD-10 mapping for research in biologics and biosimilars using administrative healthcare data. Pharmacoepidemiology and Drug Safety, 28, 210–210. doi: 10.1016/j.jval.2019.04.122 31854053

[ref21] Howard, D. M., Adams, M. J., Clarke, T. K., Hafferty, J. D., Gibson, J., Shirali, M., … McIntosh, A. M. (2019). Genome-wide meta-analysis of depression identifies 102 independent variants and highlights the importance of the prefrontal brain regions. Nature Neuroscience, 22(3), 343–352. doi: 10.1038/s41593-018-0326-7 30718901 PMC6522363

[ref22] Kendler, K. S., Gardner, C. O., & Prescott, C. A. (2002). Toward a comprehensive developmental model for major depression in women. American Journal of Psychiatry, 159(7), 1133–1145. doi: 10.1176/appi.ajp.159.7.1133 12091191

[ref23] Kendler, K. S., Gardner, C. O., & Prescott, C. A. (2006). Toward a comprehensive developmental model for major depression in men. American Journal of Psychiatry, 163(1), 115–124. doi: 10.1176/appi.ajp.163.1.115 16390898

[ref24] Kendler, K. S., Gatz, M., Gardner, C. O., & Pedersen, N. L. (2005). Age at onset and familial risk for major depression in a Swedish national twin sample. Psychological Medicine, 35(11), 1573–1579. doi: 10.1017/S0033291705005714 16219115

[ref25] Kim, W., & Chen, Y. L. (2011). The social determinants of depression in elderly Korean immigrants in Canada: Does acculturation matter? The International Journal of Aging and Human Development, 73(4), 283–298. doi: 10.2190/AG.73.4.a 22474912

[ref26] Koh, H. K., Piotrowski, J. J., Kumanyika, S., & Fielding, J. E. (2011). Healthy people: A 2020 vision for the social determinants approach. Health Education & Behavior, 38(6), 551–557. doi: 10.1177/1090198111428646 22102542

[ref27] Lee, S. H., & Kim, Y. B. (2014). Which type of social activities decrease depression in the elderly? An analysis of a population-based study in South Korea. Iranian Journal of Public Health, 43(7), 903–912. Retrieved from https://pubmed.ncbi.nlm.nih.gov/25909058/. 25909058 PMC4401055

[ref28] Li, R. W., Chen, Y., Ritchie, M. D., & Moore, J. H. (2020). Electronic health records and polygenic risk scores for predicting disease risk. Nature Reviews Genetics, 21(8), 493–502. doi: 10.1038/s41576-020-0224-1 32235907

[ref29] Liang, Y., Gong, Y. H., Wen, X. P., Guan, C. P., Li, M. C., Yin, P., & Wang, Z. Q. (2012). Social determinants of health and depression: A preliminary investigation from rural China. PLoS ONE, 7(1), e30553. doi: 10.1371/journal.pone.0030553 22276213 PMC3261904

[ref30] Litwin, H., Stoeckel, K. J., & Schwartz, E. (2015). Social networks and mental health among older Europeans: Are there age effects? European Journal of Ageing, 12(4), 299–309. doi: 10.1007/s10433-015-0347-y 28804362 PMC5549154

[ref31] Mackenzie, C. S., Reynolds, K., Cairney, J., Streiner, D. L., & Sareen, J. (2012). Disorder-specific mental health service use for mood and anxiety disorders: Associations with age, sex, and psychiatric comorbidity. Depression and Anxiety, 29(3), 234–242. doi: 10.1002/da.20911 22065571 PMC4284961

[ref32] McClintock, H. F., & Bogner, H. R. (2017). Incorporating patients’ social determinants of health into hypertension and depression care: A pilot randomized controlled trial. Community Mental Health Journal, 53(6), 703–710. doi: 10.1007/s10597-017-0131-x 28378301 PMC5511567

[ref33] Min, J., Ailshire, J., & Crimmins, E. M. (2016). Social engagement and depressive symptoms: Do baseline depression status and type of social activities make a difference? Age and Ageing, 45(6), 838–843. doi: 10.1093/ageing/afw125 27496942 PMC6312002

[ref34] Natekin, A., & Knoll, A. (2013). Gradient boosting machines, a tutorial. Frontiers in Neurorobotics, 7, 21. doi: 10.3389/fnbot.2013.00021 PMC3885826 24409142

[ref35] Olson, J. E., Ryu, E., Hathcock, M. A., Gupta, R., Bublitz, J. T., Takahashi, P. Y., … Cerhan, J. R. (2019). Characteristics and utilisation of the Mayo Clinic Biobank, a clinic-based prospective collection in the USA: Cohort profile. BMJ Open, 9(11), e032707. doi: 10.1136/bmjopen-2019-032707 PMC6858142 31699749

[ref36] Olson, J. E., Ryu, E., Johnson, K. J., Koenig, B. A., Maschke, K. J., Morrisette, J. A., … Cerhan, J. R. (2013). The Mayo Clinic Biobank: A building block for individualized medicine. Mayo Clinic Proceedings, 88(9), 952–962. doi: 10.1016/j.mayocp.2013.06.006 24001487 PMC4258707

[ref37] Park, S. C., Hahn, S. W., Hwang, T. Y., Kim, J. M., Jun, T. Y., Lee, M. S., … Park, Y. C. (2014). Does age at onset of first major depressive episode indicate the subtype of major depressive disorder?: The clinical research center for depression study. Yonsei Medical Journal, 55(6), 1712–1720. doi: 10.3349/ymj.2014.55.6.1712 25323911 PMC4205714

[ref38] Power, R. A., Tansey, K. E., Buttenschon, H. N., Cohen-Woods, S., Bigdeli, T., Hall, L. S., … Lewis, C. M. (2017). Genome-wide association for major depression through age at onset stratification: Major depressive disorder working group of the psychiatric genomics consortium. Biological Psychiatry, 81(4), 325–335. doi: 10.1016/j.biopsych.2016.05.010 27519822 PMC5262436

[ref39] Roy, M., Levasseur, M., Dore, I., St-Hilaire, F., Michallet, B., Couturier, Y., … Genereux, M. (2018). Looking for capacities rather than vulnerabilities: The moderating effect of health assets on the associations between adverse social position and health. Preventive Medicine, 110, 93–99. doi: 10.1016/j.ypmed.2018.02.014 29454078

[ref40] Santini, Z. I., Jose, P. E., Cornwell, E. Y., Koyanagi, A., Nielsen, L., Hinrichsen, C., … Koushede, V. (2020). Social disconnectedness, perceived isolation, and symptoms of depression and anxiety among older Americans (NSHAP): A longitudinal mediation analysis. Lancet Public Health, 5(1), E62–E70. doi: 10.1016/S2468-2667(19)30230-0 31910981

[ref41] Sarris, J., Thomson, R., Hargraves, F., Eaton, M., de Manincor, M., Veronese, N., … Firth, J. (2020). Multiple lifestyle factors and depressed mood: A cross-sectional and longitudinal analysis of the UK Biobank (*N* = 84860). BMC Medicine, 18(1), 354. doi: 10.1186/s12916-020-01813-5 PMC7661271 33176802

[ref42] Sekhon, S., Patel, J., & Sapra, A. (2021). Late onset depression. Retreated from https://www.ncbi.nlm.nih.gov/books/NBK551507/.

[ref43] Shah, A. D., Bartlett, J. W., Carpenter, J., Nicholas, O., & Hemingway, H. (2014). Comparison of random forest and parametric imputation models for imputing missing data using MICE: A CALIBER study. American Journal of Epidemiology, 179(6), 764–774. doi: 10.1093/aje/kwt312 24589914 PMC3939843

[ref44] Shittu, R. O., Issa, B. A., Olanrewaju, G. T., Mahmoud, A. O., Odeigah, L. O., & Sule, A. G. (2014). Social determinants of depression: Social cohesion, negative life events, and depression among people living with HIV/Aids in Nigeria, West Africa. The International Journal of Maternal and Child Health (MCH) and AIDS (IJMA), 2(2), 174–181. Retrieved from https://www.ncbi.nlm.nih.gov/pubmed/27621970. PMC4948142 27621970

[ref45] Singh, G. K., Daus, G. P., Allender, M., Ramey, C. T., Martin, E. K., Perry, C., … Vedamuthu, I. P. (2017). Social determinants of health in the United States: Addressing major health inequality trends for the nation, 1935–2016. International Journal of MCH and AIDS, 6(2), 139–164. doi: 10.21106/ijma.236 29367890 PMC5777389

[ref46] Sloan, D. M., & Sandt, A. R. (2006). Gender differences in depression. Women's Health, 2(3), 425–434. doi: 10.2217/17455057.2.3.425 19803914

[ref47] Smoller, J. W. (2018). The use of electronic health records for psychiatric phenotyping and genomics. American Journal of Medical Genetics. Part B, Neuropsychiatric Genetics, 177(7), 601–612. doi: 10.1002/ajmg.b.32548 PMC6440216 28557243

[ref48] Takahashi, P. Y., Ryu, E., Olson, J. E., Anderson, K. S., Hathcock, M. A., Haas, L. R., … Cerhan, J. R. (2013). Hospitalizations and emergency department use in Mayo Clinic Biobank participants within the employee and community health medical home. Mayo Clinic Proceedings, 88(9), 963–969. doi: 10.1016/j.mayocp.2013.06.015 24001488 PMC4151531

[ref49] Tanner, E. K., Martinez, I. L., & Harris, M. (2014). Examining functional and social determinants of depression in community-dwelling older adults: Implications for practice. Geriatric Nursing, 35(3), 236–240. doi: 10.1016/j.gerinurse.2014.04.006 24942525

[ref50] Townsend, L., Walkup, J. T., Crystal, S., & Olfson, M. (2012). A systematic review of validated methods for identifying depression using administrative data. Pharmacoepidemiology and Drug Safety, 21, 163–173. doi: 10.1002/pds.2310 22262603

[ref51] Vaglio, J. Jr., Conard, M., Poston, W. S., O'Keefe, J., Haddock, C. K., House, J., & Spertus, J. A. (2004). Testing the performance of the ENRICHD social support instrument in cardiac patients. Health and Quality of Life Outcomes, 2, 24. doi:10.1186/1477-7525-2-24 15142277 PMC434528

[ref52] Vahratian, A., Blumberg, S. J., Terlizzi, E. P., & Schiller, J. S. (2021). Symptoms of anxiety or depressive disorder and use of mental health care among adults during the COVID-19 pandemic – United States, August 2020–February 2021. MMWR Morbidity and Mortality Weekly Report, 70(13), 490–494. doi: 10.15585/mmwr.mm7013e2 33793459 PMC8022876

[ref53] Wang, J. Y., Mann, F., Lloyd-Evans, B., Ma, R. M., & Johnson, S. (2018). Associations between loneliness and perceived social support and outcomes of mental health problems: A systematic review. BMC Psychiatry, 18, 156. doi: 10.1186/s12888-018-1736-5 PMC5975705 29843662

[ref54] Wei, W. Q., Bastarache, L. A., Carroll, R. J., Marlo, J. E., Osterman, T. J., Gamazon, E. R., … Denny, J. C. (2017). Evaluating phecodes, clinical classification software, and ICD-9-CM codes for phenomewide association studies in the electronic health record. PLoS ONE, 12(7), e0175508. doi: 10.1371/journal.pone.0175508 PMC5501393 28686612

[ref55] Wi, C. I., St Sauver, J. L., Jacobson, D. J., Pendegraft, R. S., Lahr, B. D., Ryu, E., … Juhn, Y. J. (2016). Ethnicity, socioeconomic status, and health disparities in a mixed rural-urban US community-Olmsted County, Minnesota. Mayo Clinic Proceedings, 91(5), 612–622. doi: 10.1016/j.mayocp.2016.02.011 27068669 PMC4871690

