# Supplementary material for: The importance of social activity to risk of major depression in older adults
Source: Psychol Med. 2021 Nov 12;53(6):2634–42. doi: 10.1017/S0033291721004566 (PMC9095757; doi:10.1017/S0033291721004566)

**Supplemental Table 1**. A list of ICD9/10 codes used to identify older adults with major depressive disorder

| **ICD codes** | **Specific codes** | **ICD9 String** | **PheCode** |
| --- | --- | --- | --- |
| ICD9 | 296.20 | Major depressive disorder, single episode, unspecified degree | 296.22 |
| ICD9 | 296.21 | Major depressive disorder, single episode, mild degree | 296.2 |
| ICD9 | 296.22 | Major depressive disorder, single episode, moderate degree | 296.22 |
| ICD9 | 296.23 | Major depressive disorder, single episode, severe degree, without mention of psychotic behavior | 296.22 |
| ICD9 | 296.24 | Major depressive disorder, single episode, severe degree, specified as with psychotic behavior | 296.22 |
| ICD9 | 296.25 | Major depressive disorder, single episode, in partial or unspecified remission | 296.22 |
| ICD9 | 296.26 | Major depressive disorder, single episode in full remission | 296.22 |
| ICD9 | 296.30 | Major depressive disorder, recurrent episode, unspecified degree | 296.22 |
| ICD9 | 296.31 | Major depressive disorder, recurrent episode, mild degree | 296.2 |
| ICD9 | 296.32 | Major depressive disorder, recurrent episode, moderate degree | 296.22 |
| ICD9 | 296.33 | Major depressive disorder, recurrent episode, severe degree, without mention of psychotic behavior | 296.22 |
| ICD9 | 296.34 | Major depressive disorder, recurrent episode, severe degree, specified as with psychotic behavior | 296.22 |
| ICD9 | 296.35 | Major depressive disorder, recurrent episode, in partial or unspecified remission | 296.22 |
| ICD9 | 296.36 | Major depressive disorder, recurrent episode, in full remission | 296.22 |
| ICD9 | 296.82 | Atypical depressive disorder | 296.1 |
| ICD9 | 298.0 | Depressive type psychosis | 295.3 |
| ICD9 | 300.4 | Dysthymic disorder | 300.4 |
| ICD9 | 311 | Depressive disorder NEC | 296.2 |
| ICD10 | F32.0 | Major depressive disorder, single episode, mild | 296.22 |
| ICD10 | F32.1 | Major depressive disorder, single episode, moderate | 296.22 |
| ICD10 | F32.2 | Major depressive disorder, single episode, severe without psychotic features | 296.22 |
| ICD10 | F32.3 | Major depressive disorder, single episode, severe with psychotic features | 296.22 |
| ICD10 | F32.4 | Major depressive disorder, single episode, in partial remission | 296.22 |
| ICD10 | F32.5 | Major depressive disorder, single episode, in full remission | 296.22 |
| ICD10 | F32.8 | Other depressive episodes | 296.22 |
| ICD10 | F32.9 | Major depressive disorder, single episode, unspecified |  |
| ICD10 | F33.0 | Major depressive disorder, recurrent, mild | 296.22 |
| ICD10 | F33.1 | Major depressive disorder, recurrent, moderate | 296.22 |
| ICD10 | F33.2 | Major depressive disorder, recurrent severe without psychotic features | 296.22 |
| ICD10 | F33.3 | Major depressive disorder, recurrent, severe with psychotic symptoms | 296.22 |
| ICD10 | F33.40 | Major depressive disorder, recurrent, in remission, unspecified | 296.22 |
| ICD10 | F33.41 | Major depressive disorder, recurrent, in partial remission | 296.22 |
| ICD10 | F33.42 | Major depressive disorder, recurrent, in full remission | 296.22 |
| ICD10 | F33.8 | Other recurrent depressive disorders | 296.22 |
| ICD10 | F34.1 | Dysthymic disorder | 300.4 |
| ICD10 | F33.9 | Major depressive disorder, recurrent, unspecified | 296.22 |

Note that some codes indicate recurrent MDD or MDD in remission (e.g., 296.26). We did not exclude those codes because we do not have patients’ full EHR data (see the Limitation section) to reliably classify MDD subtype such as recurrent status using available data. We acknowledge that the study cohort may include participants with a prior history of MDD, even though we excluded patients with prior MDD diagnoses in EHR or self-reported history of an MDD diagnosis. However, this is a limitation of any studies utilizing EHR data, not specific to our study.

**Supplementary Table 2.** Social determinants of health (SDoH) questions used in this study

| **Variables** | **Questions** | **Categories used in the analysis** |
| --- | --- | --- |
| **Demographic characteristics** |  |  |
| Race | *Which of the following do you consider yourself?*  Asian; Black or African American; White; American Indian or Alaskan Native; Native Hawaiian or other Pacific Islander; Other | White; Others (non-Whites) |
| Hispanic | *Do you consider yourself to be Hispanic or Latino?*  Yes; No | Yes; No |
| US born | *Where you born in the United States?*  Yes; No | Yes; No |
| Marital status | *Are you currently..*  Married; living with someone in a marriage-like relationship; separated; divorced; widowed; never been married | Married; Marriage-like relationship; Separated/Divorced/Widowed/Never been married |
| Education attainment | *Which is the highest grade or level of school you have completed?*  8^th^ grade or less; some high school; high school graduate or GED; vocational, technical, or business school; some college or associate degree; four year college graduate; Graduate or professional school; other | High school or less; Some college (vocational, technical, or business school; some college or associate degree); College graduate; Graduate/professional degree |
| Employment | *Which of the following best describes you?*  Working full time for pay; working part-time for pay; not working for pay at present | Full-time; Part-time/Not working for pay |
| **Social activity and support** |  |  |
| Level of social activity | *How would you describe your level of social activity?*  0 (as bad as it can be) to 10 (as good as it can be) | Low (0-5); Medium (6-7); High (8-10) |
| Someone to listen | *How much of the time is there someone available to you whom you can count on to listen when you need to talk?*  None of the time (1); A little of the time (2); Some of the time (3); Most of the time (4); All of the time (5) | Little (None or A little); Some; Most (Most or All) |
| Someone to give advice | *How much of the time is there someone available to you to give you good advice about a problem?*  None of the time (1); A little of the time (2); Some of the time (3); Most of the time (4); All of the time (5) | Little (None or A little); Some; Most (Most or All) |
| Someone for love | *How much of the time is there someone available to you who shows you love and affection?*  None of the time (1); A little of the time (2); Some of the time (3); Most of the time (4); All of the time (5) | Little (None or A little); Some; Most (Most or All) |
| Someone for daily chore | *How much of the time is there someone available to help with daily chores?*  None of the time (1); A little of the time (2); Some of the time (3); Most of the time (4); All of the time (5) | Little (None or A little); Some; Most (Most or All) |
| Someone for emotional support | *How much of the time can you count on anyone to provide you with emotional support (talking over problems or helping you make a difficult decision)?*  None of the time (1); A little of the time (2); Some of the time (3); Most of the time (4); All of the time (5) | Little (None or A little); Some; Most (Most or All) |
| Someone to trust and confide | *How much of the time do you have as much as contact as you would like with someone you feel close to, someone in whom you can trust and confide in?*  None of the time (1); A little of the time (2); Some of the time (3); Most of the time (4); All of the time (5) | Little (None or A little); Some; Most (Most or All) |
| **General health behaviors** |  |  |
| Alcohol consumptions | *How often did you have a drink containing alcohol in the past 12 months?*  Neve; Once a month or less; 2 to 4 times per month; 2 to 3 times a week; 4 to 5 times a week; 6 or more times a week | Never/rare; 2-4 times per month; 2-5 times per week; 6 or more per week |
| Smoking | *Have you smoked at least 100 cigarettes in your entire life?*  Yes; No; Don’t know/not sure  *If yes, do you currently smoke cigarettes?*  Yes; No | Never; Past; Current |
| Regular dental check-up | *Have you seen a dentist for a general check-up and teeth cleaning within the last 12 months?*  Yes; No | Yes; No |
| High fat intake a day | *On average, how many times a day do you eat high-fat food such as red meat, fried food, whole milk, regular cheese, ice cream,, baked goods, or regular salad dressing?*  0 to 1; 2; 3 or more | 0-1; 2; 3 or more |
| Fruit servings a day | *How man servings of fruit do you eat during a typical day?*  0 to 1; 2; 3; 4; 5 or more | 0-1; 2; 3 or more |
| Vegetable servings a day | *How many servings of vegetables do you eat during a typical day?*  0 to 1; 2; 3; 4; 5 or more | 0-1; 2; 3 or more |
| **Physical activities** |  |  |
| Exercise (Godin score) | *Considering a 7-day period, how many times on average do you do the following kinds of exercise for more than 15 minutes during your free time?*   - Strenuous exercise: none;1;2;3;4;5;6;7; 8 times or more - Moderate exercise: none, 1;2;3;4;5;6;7; 8 times or more - Mild exercise; none, 1;2;3;4;5;6;7; 8 times or more | Total score  =9*[strenuous]+5*[moderate]+3*[mild]  Sedentary (<14); Moderate (14-23); High (24 or more) |
| Sitting at job | *For the job (including homemaking) you held the longest, approximately how much of the time were you engaged in “sitting”?*  None of the time; A little of the time; Some of the time; Most of the time: All of the time | Little (None or A little); Some; Most (Most or All) |
| Standing at job | *For the job (including homemaking) you held the longest, approximately how much of the time were you engaged in “standing”?*  None of the time; A little of the time; Some of the time; Most of the time: All of the time | Little (None or A little); Some; Most (Most or All) |
| Walking at job | *For the job (including homemaking) you held the longest, approximately how much of the time were you engaged in “walking”?*  None of the time; A little of the time; Some of the time; Most of the time: All of the time | Little (None or A little); Some; Most (Most or All) |
| Light labor at job | *For the job (including homemaking) you held the longest, approximately how much of the time were you engaged in “light manual labor”?*  None of the time; A little of the time; Some of the time; Most of the time: All of the time | Little (None or A little); Some; Most (Most or All) |
| Heavy labor at job | *For the job (including homemaking) you held the longest, approximately how much of the time were you engaged in “heavy manual labor”?*  None of the time; A little of the time; Some of the time; Most of the time: All of the time | Little (None or A little); Some; Most (Most or All) |
| **Environmental factors** |  |  |
| Secondhand smoking | *Did you ever live in the same household with someone who smoked cigarettes regularly while your presence?*  Yes; No | Yes; No |
| Residential location | *Where do you currently live most of the year?*  On a working farm or ranch; In a rural home or hobby farm, not a working farm or ranch; In a suburb, city, or village; Other | On a working farm/ranch; In a rural/hobby home; In a suburb/city/village; Other |
| History of living in a farm | *Have you ever lived on a working farm?*  Yes; No | Yes; No |

**Supplemental Table 3.** Summary of social determinants of health in subjects included in the full cohort and in a sub-cohort consisting of subjects who received routine medical care at Mayo Clinic

|  | Full cohort  (N=30,236) | | | Subcohort  (N=11,716) | | |
| --- | --- | --- | --- | --- | --- | --- |
| Characteristics | Summary | Hazard ratio  (95% CI) | P-value | Summary | Hazard ratio  (95% CI) | P-value |
| **Demographic characteristics** |  |  |  |  |  |  |
| Age (in years) at survey,  median (25^th^ -75^th^ %tile ) | 67 (59, 74) | -- | -- | 66 (58, 73) | -- | -- |
| Gender, n (%)  Female  Male | 15,191 (50.2%)  15,045 (49.8%) | -- | -- | 6,313 (53.9%)  5,403 (46.1%) | -- | -- |
| Race, n (%)  White  Others | 28,139 (93.1%)  2,097 (6.9%) | 0.85 (0.72, 1.00)  Ref | 0.063 | 11,014 (94.0%)  702 (6.0%) | 0.79 (0.62, 0.99)  Ref | 0.516 |
| Hispanic, n (%)  Yes  No  *Missing* | 340 (1.1%)  29,551 (97.7%)  345 (1.1%) | 1.46 (1.02, 2.09)  Ref | 0.054 | 115 (1.0%)  11,486 (98.0%)  115 (1.0%) | 1.81 (1.10, 2.96)  Ref | 0.033 |
| US born, n (%)  Yes  No  *Missing* | 28,841 (95.4%)  1,283 (4.2%)  112 (0.4%) | 1.01 (0.81, 1.25)  Ref | 0.945 | 11,229 (95.8%)  447 (3.8%)  40 (0.3%) | 0.87 (0.65, 1.17)  Ref | 0.357 |
| Marital status, n (%)  Married  Marriage-like relationship  Separated/Divorced  Widowed  Never been married  *Missing* | 24,334 (80.5%)  726 (2.4%)  1,724 (5.7%)  1,983 (6.6%)  876 (2.9%)  593 (2.0%) | Ref  1.23 (0.94, 1.62)  1.37 (1.15, 1.62)  1.24 (1.05, 1.46)  1.11 (0.87, 1.42) | < 0.001 | 9,361 (79.7%)  218 (1.9%)  678 (5.8%)  807 (6.9%)  423 (3.6%)  229 (2.0%) | Ref  1.44 (0.96, 2.16)  1.37 (1.08, 1.73)  1.10 (0.87, 1.38)  0.92 (0.66, 1.27) | 0.046 |
| Education attainment, n (%)  High school or less  Some college  College graduate  Graduate/professional degree  *Missing* | 5,431 (18.0%)  8,910 (29.5%)  7,215 (23.9%)  8,189 (27.1%)  491 (1.6%) | Ref  0.89 (0.79, 1.00)  0.62 (0.54, 0.71)  0.66 (0.58, 0.75) | < 0.001 | 2,198 (18.8%)  3,764 (32.1%)  2,771 (23.7%)  2,799 (23.9%) | Ref  0.85 (0.72, 0.99)  0.61 (0.51, 0.74)  0.68 (0.57, 0.82) | <0.001 |
| Employment, n (%)  Full-time  Part-time  Not working for pay  *Missing* | 8,981 (29.7%)  3,484 (11.5%)  17,322 (57.3%)  449 (1.5%) | Ref  1.19 (1.01, 1.40)  1.34 (1.18, 1.53) | < 0.001 | 3,767 (32.2%)  1,423 (12.1%)  6,387 (54.5%)  139 (1.2%) | Ref  1.09 (0.87, 1.37)  1.42 (1.17, 1.73) | <0.001 |
| **Social activity and support** |  |  |  |  |  |  |
| Level of social activity, n (%)  0 – 5 (Low)  6 – 7 (Medium)  8 – 10 (High)  *Missing* | 3,422 (11.3%)  6,233 (20.6%)  20,514 (67.8%)  67 (0.2%) | Ref  0.58 (0.50, 0.66)  0.44 (0.40, 0.50) | < 0.001 | 1,093 (9.3%)  2,451 (20.9%)  8,141 (69.5%)  31 (0.3%) | Ref  0.54 (0.44, 0.66)  0.42 (0.36, 0.50) | <0.001 |
| Someone to listen, n (%)  Little  Some  Most  *Missing* | 1,416 (4.7%)  2,798 (9.3%)  25,978 (85.9%)  44 (0.1%) | Ref  0.92 (0.75, 1.14)  0.65 (0.55, 0.78) | < 0.001 | 501 (4.3%)  1,084 (9.3%)  10,112 (86.3%)  19 (0.2%) | Ref  0.90 (0.66, 1.23)  0.70 (0.54, 0.91) | 0.003 |
| Someone to give advice, n (%)  Little  Some  Most  *Missing* | 1,638 (5.4%)  3,770 (12.5%)  24,778 (81.9%)  50 (0.2%) | Ref  0.82 (0.67, 0.99)  0.65 (0.55, 0.77) | < 0.001 | 582 (5.0%)  1,406 (12.0%)  9,708 (82.9%)  20 (0.2%) | Ref  0.89 (0.67, 1.18)  0.69 (0.54, 0.88) | <0.001 |
| Someone for love, n (%)  Little  Some  Most  *Missing* | 1,260 (4.2%)  1,941 (6.4%)  26,938 (89.1%)  97 (0.3%) | Ref  0.88 (0.69, 1.11)  0.69 (0.57, 0.83) | < 0.001 | 477 (4.1%)  736 (6.3%)  10,470 (89.4%)  33 (0.3%) | Ref  1.11 (0.77, 1.58)  0.86 (0.64, 1.15) | 0.078 |
| Someone for daily chore, n (%)  Little  Some  Most  *Missing* | 4,098 (13.6%)  3,775 (12.5%)  22,281 (73.7%)  82 (0.3%) | Ref  0.87 (0.74, 1.02)  0.78 (0.69, 0.88) | < 0.001 | 1,584 (13.5%)  1,465 (12.5%)  8,632 (73.7%)  35 (0.3%) | Ref  0.80 (0.64, 1.00)  0.80 (0.68, 0.95) | 0.037 |
| Someone for emotional support, n (%)  Little  Some  Most  *Missing* | 1,628 (5.4%)  2,574 (8.5%)  25,976 (85.9%)  58 (0.2%) | Ref  0.83 (0.67, 1.03)  0.68 (0.58, 0.81) | < 0.001 | 582 (5.0%)  969 (8.3%)  10,142 (86.6%)  23 (0.2%) | Ref  0.82 (0.61, 1.12)  0.70 (0.55, 0.89) | 0.010 |
| Someone to trust and confide, n (%)  Little  Some  Most  *Missing* | 1,817 (6.0%)  2,651 (8.8%)  25,703 (85.0%)  65 (0.2%) | Ref  0.89 (0.73, 1.09)  0.71 (0.61, 0.84) | < 0.001 | 667 (5.7%)  991 (8.5%)  10,033 (85.6%)  25 (0.2%) | Ref  0.87 (0.65, 1.17)  0.71 (0.57, 0.90) | 0.006 |
| **General health behaviors** |  |  |  |  |  |  |
| Alcohol consumption, n (%)  Never/rare  2-4 times per month  2-5 times per week  6 or more per week  *Missing* | 11,570 (38.3%)  6,107 (20.2%)  8,503 (28.1%)  3,899 (12.9%)  157 (0.5%) | Ref  0.87 (0.78, 0.98)  0.85 (0.77, 0.95)  0.80 (0.69, 0.93) | 0.003 | 4,432 (37.8%)  2,605 (22.2%)  3,222 (27.5%)  1,413 (12.1%)  44 (0.4%) | Ref  0.83 (0.70, 0.97)  0.89 (0.76, 1.03)  0.86 (0.70, 1.06) | 0.096 |
| Smoking, n (%)  Never  Past  Current  *Missing* | 16,635 (55.0%)  12,136 (40.1%)  906 (3.0%)  559 (1.8%) | Ref  1.24 (1.13, 1.36)  2.06 (1.68, 2.52) | <0.001 | 6,543 (55.8%)  4,559 (38.9%)  399 (3.4%)  215 (1.8%) | Ref  1.28 (1.13, 1.46)  2.18 (1.68, 2.84) | <0.001 |
| Regular dental check-up, n (%)  Yes  No  Missing | 26,582 (87.9%)  3,592 (11.9%)  62 (0.2%) | 0.69 (0.61, 0.78)  Ref | < 0.001 | 10,309 (88.0%)  1,375 (11.7%)  32 (0.3%) | 0.67 (0.57, 0.79)  Ref | <0.001 |
| High fat intake a day, n (%)  0 to 1  2  3 or more  Missing | 17,004 (56.2%)  9,841 (32.5%)  3,286 (10.9%)  105 (0.3%) | Ref  1.15 (1.05, 1.26)  1.10 (0.96, 1.27) | 0.012 | 6,664 (56.9%)  3,794 (32.4%)  1,220 (10.4%)  38 (0.3%) | Ref  1.06 (0.93, 1.21)  0.98 (0.80, 1.21) | 0.634 |
| Fruit servings a day, n (%)  0 to 1  2  3 or more  Missing | 10,020 (33.1%)  11,602 (38.4%)  8,519 (28.2%)  96 (0.3%) | Ref  0.92 (0.83, 1.02)  0.81 (0.73, 0.91) | 0.002 | 3,732 (31.9%)  4,475 (38.2%)  3,473 (29.6%)  36 (0.3%) | Ref  0.85 (0.74 0.98)  0.72 (0.62, 0.85) | <0.001 |
| Vegetable servings a day, n (%)  0 to 1  2  3 or more  *Missing* | 8,115 (26.8%)  13,056 (43.2%)  8,994 (29.7%)  71 (0.2%) | Ref  0.76 (0.69, 0.85)  0.74 (0.66, 0.82) | < 0.001 | 3,206 (27.4%)  5,003 (42.7%)  3,482 (29.7%)  25 (0.2%) | Ref  0.70 (0.60, 0.81)  0.77 (0.65, 0.90) | < 0.001 |
| **Physical activities** |  |  |  |  |  |  |
| Exercise (Godin score), n (%)  Sedentary  Moderate  High  *Missing* | 7,262 (24.0%)  4,908 (16.2%)  16,662 (55.1%)  1,404 (4.6%) | Ref  0.87 (0.77, 0.99)  0.67 (0.61, 0.75) | < 0.001 | 2,541 (21.7%)  1,948 (16.6%)  6,752 (57.6%)  475 (4.1%) | Ref  0.79 (0.66, 0.94)  0.64 (0.56, 0.74) | <0.001 |
| Sitting at job, n (%)  Little  Some  Most  *Missing* | 6,436 (21.3%)  12,162 (40.2%)  11,220 (37.1%)  418 (1.4%) | Ref  0.90 (0.81, 1.01)  0.83 (0.74, 0.93) | 0.007 | 2,629 (22.4%)  4,764 (40.7%)  4,169 (35.6%)  154 (1.3%) | Ref  0.89 (0.77, 1.04)  0.82 (0.70, 0.96) | 0.056 |
| Standing at job, n (%)  Little  Some  Most  *Missing* | 8,402 (27.8%)  14,749 (48.8%)  6,578 (21.8%)  507 (1.7%) | Ref  1.03 (0.92, 1.14)  1.13 (1.00, 1.28) | 0.113 | 3,223 (27.5%)  5,574 (47.6%)  2,747 (23.4%)  172 (1.5%) | Ref  1.03 (0.89, 1.20)  1.18 (1.00, 1.40) | 0.107 |
| Walking at job, n (%)  Little  Some  Most  *Missing* | 9,275 (30.7%)  15,185 (50.2%)  5,275 (17.4%)  501 (1.7%) | Ref   - 1. (0.97, 1.19)   1.28 (1.13, 1.45) | < 0.001 | 3,495 (29.8%)  5,871 (50.1%)  2,171 (18.5%)  179 (1.5%) | Ref  1.06 (0.92, 1.23)  1.29 (1.09, 1.54) | 0.012 |
| Light labor at job, n (%)  Little  Some  Most  *Missing* | 14,780 (48.9%)  9,823 (32.5%)  5,021 (16.6%)  612 (2.0%) | Ref  1.14 (1.03, 1.25)  1.28 (1.14, 1.44) | < 0.001 | 5,476 (46.7%)  3,900 (33.3%)  2,121 (18.1%)  219 (1.9%) | Ref  1.17 (1.02, 1.35)  1.26 (1.07, 1.48) | 0.009 |
| Heavy labor at job, n (%)  Little  Some  Most  *Missing* | 23,918 (79.1%)  3,933 (13.0%)  1,400 (4.6%)  985 (3.3%) | Ref  1.11 (0.98, 1.26)  1.41 (1.17, 1.70) | 0.001 | 9,273 (79.1%)  1,513 (12.9%)  565 (4.8%)  365 (3.1%) | Ref  1.13 (0.94, 1.35)  1.50 (1.17, 1.92) | 0.006 |
| **Environmental factors** |  |  |  |  |  |  |
| Secondhand smoking, n (%)  Yes  No  Missing | 18,835 (62.3%)  11,317 (37.4%)  84 (0.3%) | 1.10 (1.00, 1.20)  Ref | 0.039 | 7,267 (62.0%)  4,418 (37.7%)  31 (0.3%) | 1.18 (1.04, 1.34)  Ref | 0.009 |
| Residential location, n (%)  On a working farm/ranch  In a rural/hobby home  In a suburb/city/village  Other  *Missing* | 1,653 (5.5%)  5,128 (17.0%)  22,788 (75.4%)  462 (1.5%)  205 (0.7%) | Ref  1.14 (0.92, 1.40)  1.05 (0.87, 1.27)  0.79 (0.51, 1.22) | 0.200 | 538 (4.6%)  1,852 (15.8%)  9,117 (77.8%)  139 (1.2%)  70 (0.6%) | Ref   - 1. (0.75, 1.37)   0.92 (0.70, 1.21)  0.89 (0.48, 1.62) | 0.655 |
| History of living in a farm, n (%)  Yes  No  *Missing* | 11,414 (37.7%)  18,662 (61.7%)  160 (0.5%) | 1.03 (0.95, 1.13)  Ref | 0.467 | 4,719 (40.3%)  6,943 (59.3%)  54 (0.5%) | 1.01 (0.90, 1.15)  Ref | 0.828 |

**Supplementary Table 4.** Interaction p-values between perceived level of social activity and other social determinants of health in the full cohort and in a sub-cohort consisting of subjects who received routine medical care at Mayo Clinic

|  | Full cohort  (n=30,236) | Subcohort  (11,716) |
| --- | --- | --- |
| **Demographic characteristics** |  |  |
| Age | 0.422 | 0.208 |
| Gender | 0.356 | 0.429 |
| Race | 0.824 | 0.897 |
| Hispanic | 0.376 | 0.806 |
| US born | 0.057 | 0.018 |
| Marital status | 0.400 | 0.096 |
| Education attainment | 0.071 | 0.036 |
| Employment | 0.467 | 0.598 |
| **Social support** |  |  |
| Someone to listen | 0.723 | 0.956 |
| Someone to give advice | 0.083 | 0.282 |
| Someone for love | 0.552 | 0.516 |
| Someone for daily chore | 0.746 | 0.232 |
| Someone for emotional support | 0.866 | 0.185 |
| Someone to trust and confide | 0.754 | 0.644 |
| **General health behaviors** |  |  |
| Alcohol consumption | 0.858 | 0.965 |
| Smoking | 0.490 | 0.592 |
| Regular dental check-up | 0.154 | 0.292 |
| High fat intake a day | 0.693 | 0.626 |
| Fruit servings a day | 0.371 | 0.353 |
| Vegetable servings a day | 0.089 | 0.233 |
| **Physical activities** |  |  |
| Exercise | 0.050 | 0.104 |
| Sitting at job | 0.891 | 0.864 |
| Standing at job | 0.946 | 0.706 |
| Walking at job | 0.199 | 0.565 |
| Light labor at job | 0.807 | 0.565 |
| Heavy labor at job | 0.834 | 0.761 |
| **Environmental factors** |  |  |
| Secondhand smoking | 0.738 | 0.344 |
| Residential location | 0.084 | 0.483 |
| History of living in a farm | 0.370 | 0.519 |

**Supplementary Figure 1.** Relative importance of social determinants of health variables for new episode of MDD among Mayo Clinic Biobank participants (the left panel) and a sub-cohort of participants who received routine medical care at Mayo Clinic (the right panel)


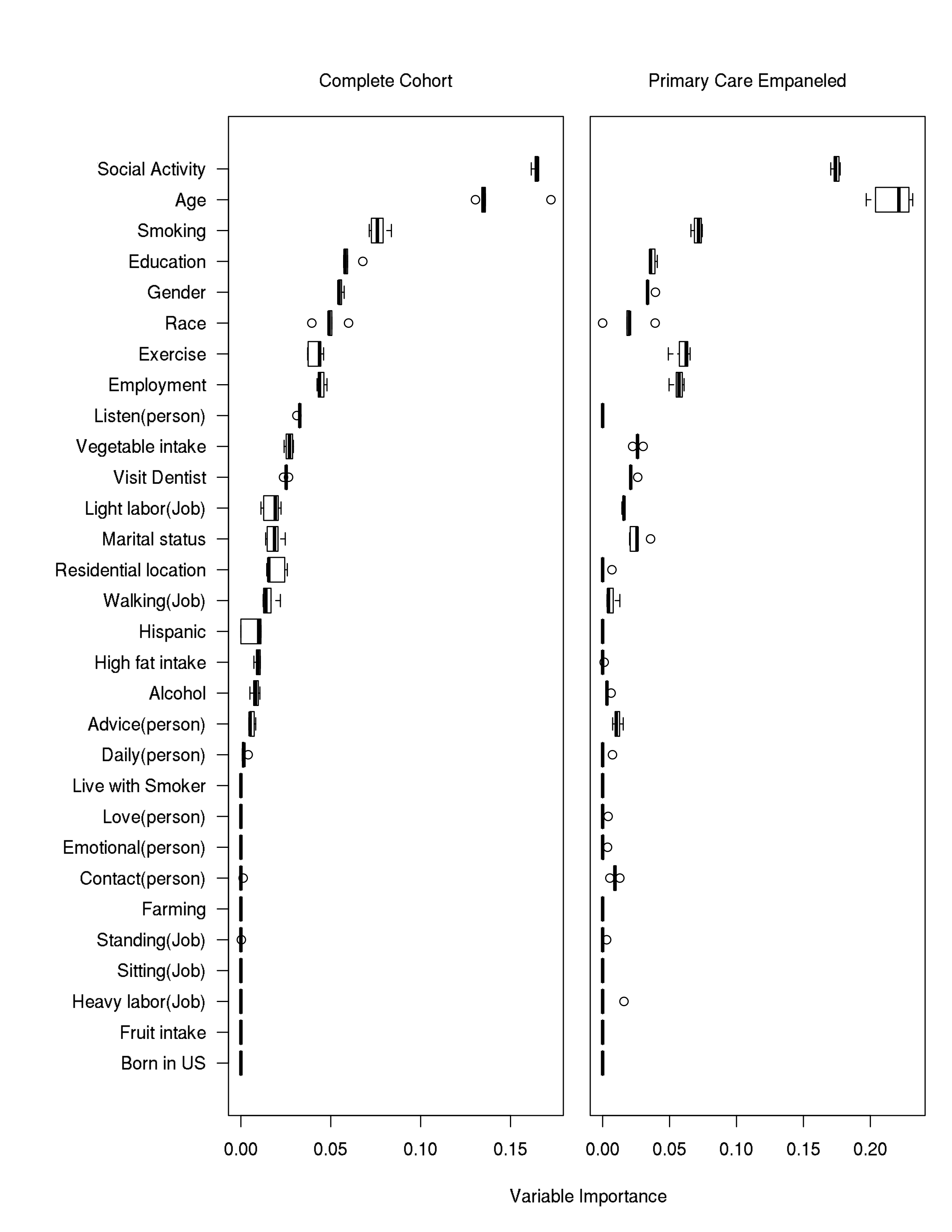


**Supplementary Figure 2.** Relationship of age and perceived level of social activity on risk of MDD in the cohort consisting of participants who received routine medical care at Mayo Clinic


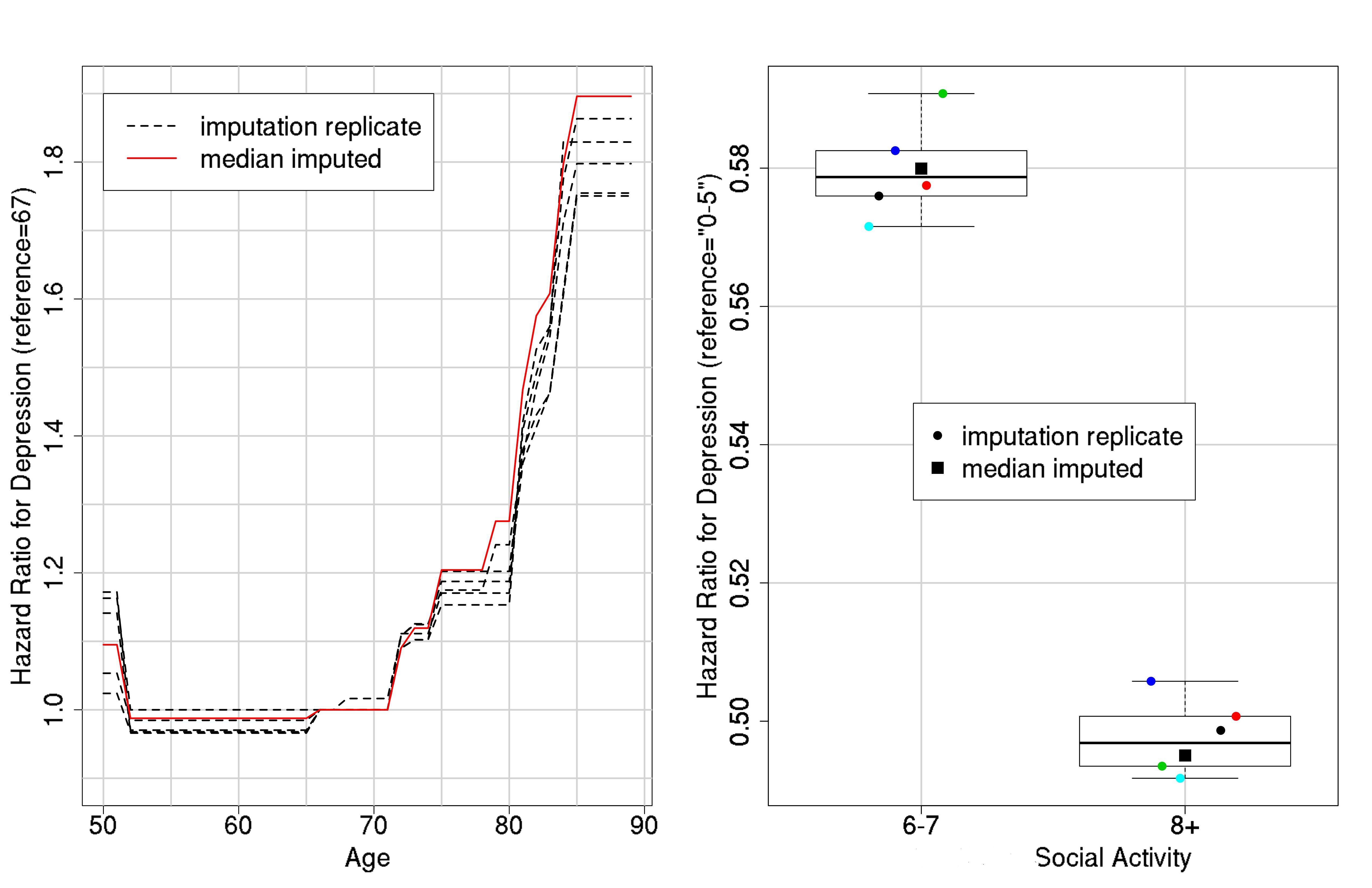

Supplement: Supplementary file 1 [file S0033291721004566sup001.docx]
